# Signaler–receiver–eavesdropper: Risks and rewards of variation in the dominant frequency of male cricket calls

**DOI:** 10.1002/ece3.6866

**Published:** 2020-09-29

**Authors:** Olivia L. Dobbs, Janelle B. Talavera, Sarina M. Rossi, Stephanie Menjivar, David A. Gray

**Affiliations:** ^1^ Department of Biology California State University Northridge Northridge CA USA

**Keywords:** coevolution, eavesdropping, sexual selection

## Abstract

Signals are important for communication and mating, and while they can benefit an individual, they can also be costly and dangerous. Male field crickets call in order to attract female crickets, but gravid females of a parasitoid fly species, *Ormia ochracea*, are also attracted to the call and use it to pinpoint male cricket hosts. Conspicuousness of the call can vary with frequency, amplitude, and temporal features. Previous work with this system has only considered temporal variation in cricket calls, both large scale, that is, amount of calling and at what time of evening, and small scale, that is, aspects of chirp rate, pulse rate, and numbers of pulses per chirp. Because auditory perception in both crickets and flies relies on the matching of the peak frequency of the call with the peripheral sensory system, peak frequency may be subject to selection both from female crickets and from female flies. Here, we used field playbacks of four different versions of the same male *Gryllus lineaticeps* calling song that only differed in peak frequency (3.3, 4.3, 5.3, and 6.3 kHz) to test the relative attractiveness of the calls to female crickets and female flies. Our results clearly show that lower frequency calls enhance male safety from fly parasitism, but that the enhanced safety would come at a cost of reduced attraction of female crickets as potential mates. The results imply that eavesdropper pressure can disrupt the matched coevolution of signalers and receivers such that the common concept of matched male–female signaler–receiver coevolution may actually be better described as male–female–predator signaler–receiver–eavesdropper coevolution.

## Graphical abstract

Male–female mating signal coevolution can be disrupted by trade‐offs with eavesdropping parasitoids.

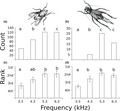

## 1 INTRODUCTION

Classical models of sexual selection posit net stabilizing selection on male signal traits due to countervailing forces of natural and sexual selection (Kirkpatrick, 1982; Lande, 1981). That is, there is often a trade‐off between the cost of a mating signal trait and the benefit of that trait for increased reproduction (e.g., Heinen‐Kay et al., 2014). Net stabilizing selection can be disrupted by changes to the strength of natural and/or sexual selection: Male signal traits can become more elaborated under low predation regimes (e.g., Endler, 1980) or conversely they can be lost entirely with dramatically increased predation pressure (e.g., Heinen‐Kay & Zuk, 2019; Zuk et al., 2006). Males may also change how much they signal (Cade, 1984) and/or when they signal (Bertram et al., 2004) in response to predators.

For some time, it has been appreciated that male sexual signals typically match the sensory‐neural capabilities of conspecific females (e.g., Gerhardt, 1994) and may evolve to match those capabilities (Endler & Basolo, 1998). Comparatively little work has considered how male signal traits match the sensory‐neural capabilities of potential “eavesdropping” predators (but see Fugère et al., 2015; Rosenthal et al., 2001), and how evolutionary changes to the male signals might increase or decrease conspicuousness to those predators. Evolution of male sexual signal conspicuousness away from the peak sensory‐neural capabilities of eavesdroppers might decrease risk, but might also entail a cost in terms of attraction of mates. This will be especially true if the sensory systems of eavesdroppers and conspecific females process male sexual signals using similarly designed peripheral and/or central filtering mechanisms. Here we explore this question with field experiments manipulating the peak frequency of cricket calls to assess the impact of call dominant frequency on attraction of female crickets and of a deadly parasitoid fly, *Ormia ochracea*.

Gravid adult female *O. ochracea* find their cricket hosts by eavesdropping on the calls of male crickets (Cade, 1975; Walker & Wineriter, 1991; Zuk et al., 1995). Flies deposit larvae on or near the host (Adamo et al., 1995). The larvae develop within the host for about 10 days before emerging to pupate, which invariably kills the cricket host (Adamo et al., 1995; Cade, 1975; Wineriter & Walker, 1990). A number of studies have shown that female flies and female crickets prefer similar temporal features of male cricket calls. This has been shown for chirp rate and duration in *Gryllus lineaticeps* (Wagner, 1996); numbers of pulses per trill in *Gryllus texensis* (Gray & Cade, 1999); and chirp rate in *Gryllus staccato* [a.k.a. “G15”: flies, Sakaguchi and Gray (2011); crickets, Hennig et al. (2016)]. Different populations of flies prefer the call of their locally most common host species (Gray et al., 2007), but less so when regularly exploiting multiple hosts (Sakaguchi & Gray, 2011). Together these results suggest a high degree of specialization of the fly to the signals of their hosts, with variation among fly populations utilizing different host species (Gray et al., 2019; Paur & Gray, 2011a).

**FIGURE 1:**
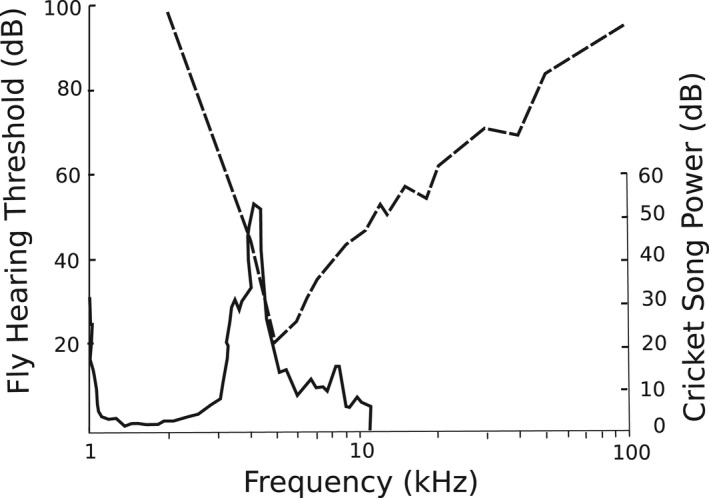
Matching between female *Ormia ochracea* hearing thresholds (left axis, dashed line) and the power spectrum of male *Gryllus* cricket calling song (right axis, solid line; here *Gryllus rubens*). Redrawn from Robert et al., 1992

The mechanics and neurobiology that underlie how the female flies acoustically locate their hosts have been studied in some detail (Lee et al., 2009; Mason & Faure, 2004; Mason et al., 2001; Miles et al., 1995; Robert et al., 1992, 1996). The peak frequency sensitivity of female *O. ochracea* hearing is closely centered around 5 kHz (Robert et al., 1992); which generally matches frequencies of their cricket host calling songs, typically 4–5 kHz (Gray et al., 2019; Weissman & Gray, 2019). But the match is not perfect and appears asymmetrical. Based on Robert et al. (1992), the auditory threshold of *O. ochracea* is ca. 60 dB at 3 kHz, ca. 40 dB at 4 kHz, ca. 20 dB at 5 kHz, and ca. 25 dB at 6 kHz (Figure 1), suggesting that the fly response to cricket calls in the 3.5 – 4.5 kHz range may be less than their response to cricket calls in the 5.5 – 6.5 kHz range. This would be consistent with the known distribution of the best frequencies of individual auditory afferent neurons in *O. ochracea*, typically 4–9 kHz (Oshinsky & Hoy, 2002), and set the stage for selection by flies to favor crickets with lower‐pitched calls (by differentially killing crickets with higher pitched calls). The goal of the work reported here was to use field playbacks of cricket calling songs that vary in peak frequency, but are otherwise identical, in order to test if such a frequency shift toward lower frequency calls would decrease risk of attack from flies. That is, we were interested in measuring the potential effects of frequencies beyond the range of current phenotypic variation, not measuring current selection within the current range of phenotypic variation. Selection may only favor such a shift, however, if it did not come at the expense of decreased attraction of female crickets, therefore we used field playbacks to simultaneously measure attraction of gravid female *O. ochracea* and female crickets.

## 2 METHODS

### 2.1 Stimuli for playback

The *G. lineaticeps* calls used in this study were modified from a *G. lineaticeps* synthetic call used by Gray et al. (2007). *Gryllus lineaticeps* typical calling song is a 6–8 pulse chirp with peak frequency between ca. 4.8 and 5.3 kHz; pulse rate varies with temperature but is about 80 p/s at 27°C (Hennig et al., 2016; Maskell, 1975; Wagner, 1996; Weissman & Gray, 2019). We used the “Change Pitch” audio feature within Audacity v2.2.1 (www.audacityteam.org) to modify the peak frequency of a synthetic standard calling song of 4.8 kHz, 80 p/s, (9 ms pulse and 3.5 ms inter‐pulse), 8 pulse/chirp (=96.5 ms chirp duration), 220 ms inter‐chirp interval, (=3.2 chirps/s) to create otherwise identical 3.3, 4.3, 5.3, and 6.3 kHz versions. It is important to note that the “Change Pitch” feature does not affect the temporal characteristics of the songs; that is, higher pitched is not “sped‐up” and lower pitched is not “slowed‐down.” Each call exemplar was saved as a.wav format file with 44.1 kHz sample rate and 16‐bit resolution.

A comment regarding playback experiments and pseudoreplication: Here, we used a single synthetic exemplar per frequency. Our view is that our technique of creating synthetic stimuli avoids the well‐known playback pseudoreplication problem, which has a long history of controversy (see McGregor, 2013). Briefly, we duplicated a single synthetically produced species‐typical digital sound file four times, and then adjusted the frequency of each sound file without affecting temporal characters. This should result in four identical song files, with species‐typical values, which differ only in frequency, thereby allowing strong inference that any differences in fly or cricket attraction are causally related to differences in frequency. Some have argued that synthetic stimuli do not necessarily solve potential pseudoreplication playback issues (see Powell & Rosenthal, 2017) as even synthetic stimuli could incorporate unknown and unintended variation among exemplars. Especially in reference to the video playbacks discussed by Powell & Rosenthal, this concern may have validity, however in the case of duplication of a single species‐typical audio file, as done here, the introduction of unknown and unintended variation among exemplars is equivalent to “demonic intrusion” as discussed by Hurlbert (1984) in his classic treatise on pseudoreplication; if “demonic intrusion” occurs, then no number of different synthetic exemplars could solve the problem.

We did not create different exemplars for different temperatures because temperature has only a trivial effect on frequency, about 40 Hz/°C which is less than among‐male variation at a given temperature (see Martin et al., 2000), and our test stimuli span 4 kHz, or 100× as much. Based on a nearby weather station [www.wunderground.com station KCAMALIB52, ~2.7 km from our site, with data in 5‐min increments during our playback dates and times, adjusted for elevation and exposure using stations KCACALAB49 and KCACALAB54 (~1.7 and ~0.6 km from our site, respectively), estimated playback temperatures varied between about 20.3–30.1°C (total range), with most playbacks at temperatures estimated between 22.3–29.2°C (range of nightly means), slightly lower than the 27°C design of our standard playback. Playing a 27°C call at ambient temperatures slightly less than 27°C could increase cricket and fly attraction based on chirp rate (because it results in chirp rates faster than average for the ambient temperature and both crickets and flies prefer faster than average chirp rates, see Wagner, 1996) but could decrease attraction based on pulse rate if there is strong discrimination against higher than normal pulse rates. Any such effect should not bias our results with respect to frequency, however, because the central processing of temporal features is mechanistically distinct from, and unlikely to be correlated with, the peripheral processing of frequency (see Baker et al., 2019; Hennig et al., 2014; Mason & Faure, 2004; Schöneich, 2020; Schöneich et al., 2015).

### 2.2 Field playbacks

All playbacks were conducted in semi‐natural open space in the Santa Monica Mountains of Los Angeles County, California (34.10, −118.71). We played the four stimuli from speakers placed underneath “slit‐traps” specially designed for *Ormia* (modified from Walker, 1989). The traps were placed at each corner of a 5 × 5 m square grid; which song was played from which of the four corners of the grid was pseudo‐randomized across replicate nights to avoid confounding frequency and location. Two such grids, 145 m apart, were used on two nights (=4 replicates) and one grid was used on 4 other nights (=4 more replicates). Starting at sunset and continuing for 2.5–3 hr per replicate, we broadcast the four calls simultaneously. Each call was broadcast at 90 dB SPL at 1 m distance, measured with a RadioShack 33–2,055 sound level meter (fast C‐weighting) and a 5‐kHz constant pure tone matched to peak amplitude of the playback stimuli. To maximize flies and crickets attracted we chose 90 dB SPL at 1 m distance in order to be at the very upper end of natural call amplitudes, for example, 85 dB SPL *Gryllus bimaculatus* (Simmons, 1988); 95 dB SPL (2 *SD* above mean) for *G. texensis* (Cade, 1979). We then continuously monitored the traps and caught and recorded the number of flies that landed on or were within each trap, and we caught and recorded the numbers of female crickets that approached within ~1 m of each trap. Flies and crickets were released at the end of each night; given that prior mark–recapture work with this population marked 1,060 flies and recaptured just 69 (Paur & Gray, 2011a), we consider the likelihood of recaptured flies biasing our results to be very low. The experiment was replicated a total of eight times over the course of seven weeks in September 2018 and September and October 2019; given the temporal separation of trials, and the abundance of flies and crickets at this location (Paur & Gray, 2011b) we expected and observed no decrease in numbers of flies or crickets caught over time. Within a replicate, all playback equipment was identical; however, playback equipment differed across replicates: Three replicates used Altec‐Lansing The Jacket H2O speakers with Sony CD Walkman D‐EJ100 players; three replicates used RadioShack AMX‐15 speakers with Sony CD Walkman D‐EJ100 players; and two replicates used Altec‐Lansing VS2320 speakers with playback from MacBook Pro laptop computers. One advantage of the analysis by ranks within replicates (see below) is that it controls for any such differences across replicates.

### 2.3 Data analyses

Because the numbers of flies and crickets can be highly variable among playback nights, and because we used different playback equipment in different replicates, we analyzed the data both by total count of individuals captured at each call (via chi‐square) and by each call's rank within a replicate averaged across replicates (one‐way ANOVA on mean ranks, allowing for tied ranks), for a similar approach, see also Gray and Cade (1999). The rationale here is that we could pool data, ignoring replicate structure, and via chi‐square test if the total numbers of flies or crickets deviated from an expectation of random distribution among the four stimuli in a global sense. Such a test could be distorted, however, by certain replicates attracting more flies or crickets than others, or among replicate variation in temperature, duration, or anything else that happened to vary among replicates. Treating each replicate as a separate event controls for variation in these externalities; doing so by ranks within each replicate reflects high levels of variation in numbers caught each night (range 19–104 flies and 0–10 crickets). Given that some readers may find our ANOVA on ranks within replicates a somewhat novel approach to data analysis, we present all of the raw data in Appendix S1 thereby allowing interested readers to explore our approach and/or employ alternative analysis methods.

We followed global tests with post hoc pairwise comparisons among calls, with manual sequential Holm–Bonferroni (Holm, 1979) adjustment for multiple tests.

### 2.4 Ethics statement

This work adheres to the ASAB/ABS Guidelines for the Use of Animals in Research, the legal requirements of the United States of America, and all institutional guidelines.

## 3 RESULTS

**FIGURE 2:**
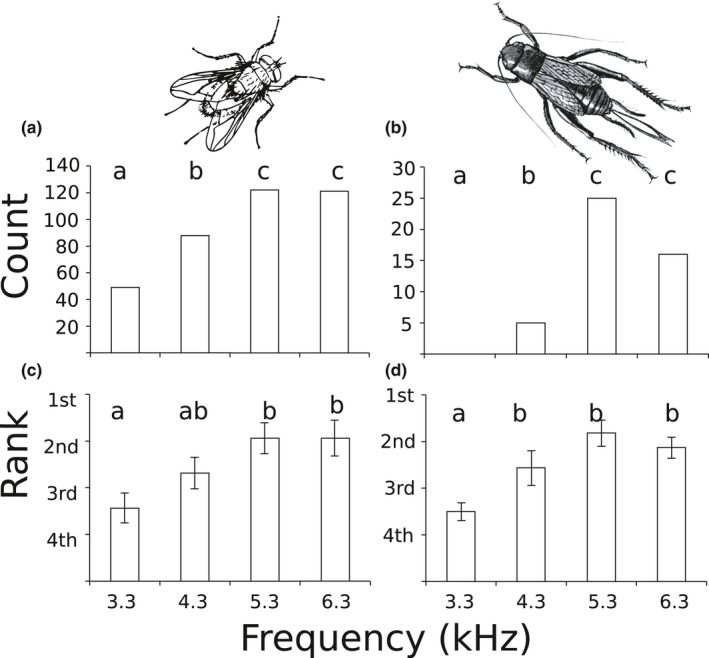
Numbers of female flies (a) and female crickets (b) caught at each of four playbacks that differed only in peak frequency of the male cricket call; (c and d) show the same results but as mean ± SE rank of each playback stimulus across *N* = 8 replicates. Counts (a, b) or ranks (c, d) with different letters are significantly different from each other

Throughout the duration of the experiment, a total of 380 female flies and 46 female crickets were caught. Both were more likely to be captured at higher pitched calling songs (Figure 2). Treating individual flies and crickets as independent replicates gave highly statistically significant results (Figure 2a,b flies: chi‐square = 37.58, *df* = 3, *p* < .00001; crickets: chi‐square = 32.78, *df* = 3, *p* < .00001). Treating the 8 individual nights as replicates, and ranking the four calls within each night (allowing for ties) also gave statistically significant results (Figure 2c,d flies: *F*_3,28_ = 4.34, *p* < .012; crickets: *F*_3,28_ = 7.08, *p* < .001). Post hoc Holm–Bonferroni tests showed that 3.3 kHz was least attractive to both female flies and female crickets by all analyses; 4.3 kHz was less attractive than either 5.3 or 6.3 kHz by total count, but not significantly so by rank.

## 4 DISCUSSION

The results of this experiment indicate that both female flies and female crickets have a preference toward higher frequency calls within the range of variation tested. Both discriminated strongly against 3.3 kHz and moderately against 4.3 kHz calls. Based upon what is known of the sensory and neural processing of sound signals by both crickets and flies, this “preference” is not due to central neural processing, but rather to the best response frequencies of the peripheral sensory system (Kostarakos et al., 2009; Mason & Faure, 2004; Michelsen et al., 1994; Oshinsky & Hoy, 2002). This indicates that male *G. lineaticeps* which call with low peak frequencies can expect reduced fitness via intraspecific sexual selection but increased fitness via decreased risk of attack by *Ormia* parasitoids.

Whether or not one selective pressure is stronger than the other would require further experimentation, but it is interesting to note that in several *Gryllus* species which face moderate or high levels of parasitism by *O. ochracea*, the peak frequency of male calls is slightly lower than the peak frequency preferences of female crickets. This is the case in *Gryllus rubens*, *G. texensis*, and *Gryllus regularis* (Blankers et al., 2015, see their Figure 2a in which *G. regularis* was studied under the name “G14”), and also in *Gryllus longicercus* (Gray et al., 2016, see their Figure 1a in which *G. longicercus* was studied under the name “G13”), potentially suggesting that male crickets have shifted their calls away from the peak sensitivity of fly hearing in several commonly utilized host species. However, the comparative evidence for this idea becomes less clear when considering additional species. For example, *Gryllus personatus* males also call with slightly lower frequencies than females most prefer (Hennig et al., 2016, their Figure 2c); *G. personatus* is a confirmed host of *O. ochracea*, but parasitism rate is low or not known throughout most of its range (Gray et al., 2019; Weissman & Gray, 2019) making the case of *G. personatus* difficult to interpret. In contrast, *G. lineaticeps*, *G. staccato*, and *Gryllus firmus* males call with peak frequencies that more closely match female peak frequency preferences (Gray et al., 2016, see their Figure 1b; Hennig et al., 2016, see their Figure 2c in which *G. staccato* was studied under the name “G15”). *Gryllus firmus* is a confirmed host of *O. ochracea* but parasitism is rare (Walker & Wineriter, 1991), which would be consistent with this idea. However, all populations of *G. staccato* likely face moderate or high levels of parasitism by *O. ochracea* (Gray et al., 2019; Weissman & Gray, 2019), so *G. staccato* is a clear counter‐example. *Gryllus lineaticeps* would also appear to be a counter‐example; however, the population of *G. lineaticeps* studied here appears to face exceptionally high levels of parasitoid pressure (Paur & Gray, 2011b), which is atypical for most populations of this species (Beckers & Wagner, 2012), and therefore, it could be the case that gene flow from low‐parasitoid populations maintains *G. lineaticeps* peak frequency higher than optimal in this population.

Evolutionary change in male song frequency is not only affected by female and/or parasitoid response, however. There are potentially other bioacoustic and life‐history/physiological trade‐offs that may constrain male signal frequency: Lower frequency sounds radiate with less power, but higher frequency sounds are more subject to reflection (Forrest, 1991; Michelsen, 1992). In crickets, lower frequency sounds require larger resonators, and therefore typically larger body size (see, e.g., Brown et al., 1996; Deb et al., 2012). Other factors such as environmental noise also may disrupt acoustic communication (Costello & Symes, 2014) and can also affect eavesdropping parasitoids (Phillips et al., 2019). Interspecific competition for acoustic sound space likewise could shift species communication systems, although evidence to date does not support this in crickets (Schmidt et al., 2016).

The observed bias in *O. ochracea* response toward higher frequencies likely reflects an evolutionary history of parasitism of hosts with higher frequency calls. All Ormiines use phonotaxis to locate Orthopteran hosts (Lehmann, 2003), but relatively little comparative work on the fly auditory system has been conducted (see Edgecomb et al., 1995; Lakes‐Harlan & Lehmann, 2015; Robert et al., 1996). All genera of Ormiines are parasitoids of katydids (Tettigoniidae) as are all known species within the genus *Ormia* except for two (Sabrosky, 1953) [note: Arnaud (1978) lists Tabanid fly larvae as an *Ormia* host, based on a single record in Jones and Anthony (1964, p. 56), but this remains unverified]. The two exceptions within *Ormia* are *O. ochracea* (cricket hosts, Gryllinae) and *O. depleta* (mole cricket hosts, Gryllotalpidae). Just from parsimony, katydids are therefore the most likely hosts to which Ormiine ears evolved (Lehmann, 2003). Katydid acoustic calls typically have much higher frequency sound energy than either crickets or mole crickets (Greenfield, 1997), therefore it seems likely that Ormiine ears evolved first to higher frequency sounds, and then subsequently to the lower frequency sounds of crickets and mole crickets. If this is the case, it is indeed likely that the preference for higher frequencies by *O. ochracea* could be partially due to an evolutionary holdover due to previous parasitism of katydids.

The observed bias in female cricket response is also interesting in that females might be expected to benefit from phonotaxis toward larger males with lower frequency calls. Nonexclusive mechanisms for this potential benefit could include direct or indirect benefits of mating with larger males (Brown et al., 1996; Simmons, 1987) and female avoidance of males more likely to be parasitized by flies (see Martin & Wagner, 2010). That female crickets of this species do not prefer lower frequency calls (see Hennig et al., 2016) may relate to constraints on directional localization of lower frequencies (Michelsen & Löhe, 1995) and/or to a history of low parasitoid pressure in most populations of this species (Beckers & Wagner, 2012).

It is also noteworthy that the numbers of female crickets attracted (46 total) was much lower than the numbers of flies attracted (380 total). As noted above, this population of crickets seems to be under exceptionally high levels of parasitoid pressure (Paur & Gray, 2011b). In that study, Paur & Gray noted that the super abundance of flies in September likely has its origin in parasitized males which had been calling in late July and early August. Calling males during the peak of fly activity at this site are extremely rare. To us it seems likely that in this population of this species of cricket, males have not reduced their calling effort or capability, which is in contrast to other species, for example, *G. texensis* in Texas (Cade, 1991) or *Teleogryllus oceanicus* in Hawaii which has lost the ability to call entirely (Zuk et al., 2006). Instead, Paur and Gray (2011b) conclude that the July/August adult males call, mate, and become parasitized by a modest population of gravid female flies, their bodies becoming the nursery for the larvae which then mature to be the massive population of late season flies. If this is correct, then it explains why the fly abundance in late fall is so much higher than the female cricket abundance. High levels of calling despite parasitism risk have also been suggested for chorusing species of Tettigoniids and Cicadas, which face acoustic parasitoids eavesdropping on male calls (Lehmann & Lakes‐Harlan, 2019). As noted by Lehman & Lakes‐Harlan, to the extent that increased pressure from eavesdropping parasitoids reduces the likelihood of future reproduction, it selects for a life history favoring early reproduction—a “call loud, die young” strategy.

Our results also relate more generally to the long‐running debate over the role of “sensory bias” in sexual selection (Ryan & Cummings, 2013). It seems generally accepted that “preferences” result from biases in the sensory and neural processing of signals (Reichert et al., 2017; Sandkam et al., 2015; Servedio & Boughman, 2017); the key issue is whether or not such bias is “pre‐existing” and drives the subsequent evolution of signals or if a more strictly coevolutionary process controls matched evolution of signals and preferences. Here, we add a further complication: A likely pre‐existing sensory bias in a predator's sensory system may drive male mating signals away from what would otherwise be the optimum reached by coevolution between males and females. The common concept of matched male–female signaler–receiver coevolution may actually be better described as male–female–predator signaler–receiver–eavesdropper coevolution. Here, we have discussed this dynamic within the communication network of conspecific male–female mating signals subject to parasitoid eavesdroppers, but we note that similar effects may apply to other communication networks such as nestling‐parent begging behaviors subject to predatory eavesdroppers (Magrath et al., 2010). At least from the eavesdropper point of view, the nature of the communications exploited (e.g., mating, begging) may well be irrelevant, potentially making the coevolutionary signaler–receiver–eavesdropper dynamic fairly widespread and applicable to multiple sensory modalities in different taxa (acoustic, visual, etc.).

## CONFLICT OF INTEREST

The authors declare that they have no conflicts of interest in publication of this article.

## AUTHOR CONTRIBUTION

**Olivia L Dobbs:** Investigation (equal); Writing‐original draft (lead); Writing‐review & editing (equal). **Janelle B Talavera:** Investigation (equal); Writing‐review & editing (supporting). **Sarina M Rossi:** Investigation (equal); Writing‐review & editing (supporting). **Stephanie Menjivar:** Investigation (equal); Supervision (supporting); Writing‐review & editing (supporting). **David Gray:** Conceptualization (lead); Data curation (lead); Formal analysis (lead); Investigation (equal); Writing‐original draft (equal); Writing‐review & editing (equal).

## Supporting information

Appendix S1

## Data Availability

The data are provided in Appendix S1.
