# Supplementary material for: Signaler–receiver–eavesdropper: Risks and rewards of variation in the dominant frequency of male cricket calls
Source: Ecol Evol. 2020 Sep 29;10(21):12364–71. doi: 10.1002/ece3.6866 (PMC7663976; doi:10.1002/ece3.6866)
Supplement: Supplementary file 1 — Appendix S1 [file ECE3-10-12364-s001.docx]

Appendix 1. Data presented by count of female flies and female crickets per replicate and by rank within replicate.

|  | **Female Flies** | |  |  | **Female Crickets** | |  |  |
| --- | --- | --- | --- | --- | --- | --- | --- | --- |
| **Replicate** | **3300 Hz** | **4300 Hz** | **5300 Hz** | **6300 Hz** | **3300 Hz** | **4300 Hz** | **5300 Hz** | **6300 Hz** |
|  |  |  |  |  |  |  |  |  |
|  | **BY COUNT** |  |  |  |  |  |  |  |
| 1 | 0 | 3 | 8 | 8 | 0 | 0 | 4 | 2 |
| 2 | 2 | 10 | 4 | 11 | 0 | 1 | 6 | 2 |
| 3 | 31 | 24 | 33 | 16 | 0 | 1 | 3 | 3 |
| 4 | 0 | 15 | 21 | 14 | 0 | 2 | 0 | 0 |
| 5 | 13 | 0 | 10 | 17 | 0 | 0 | 5 | 5 |
| 6 | 0 | 0 | 15 | 16 | 0 | 0 | 2 | 3 |
| 7 | 0 | 32 | 8 | 20 | 0 | 1 | 5 | 1 |
| 8 | 3 | 4 | 23 | 19 | 0 | 0 | 0 | 0 |
|  |  |  |  |  |  |  |  |  |
|  |  |  |  |  |  |  |  |  |
|  | **BY RANK WITHIN REPLICATE** | | |  |  |  |  |  |
| 1 | 4 | 3 | 1.5 | 1.5 | 3.5 | 3.5 | 1 | 2 |
| 2 | 4 | 2 | 3 | 1 | 4 | 3 | 1 | 2 |
| 3 | 2 | 3 | 1 | 4 | 4 | 1 | 2.5 | 2.5 |
| 4 | 4 | 2 | 1 | 3 | 3 | 1 | 3 | 3 |
| 5 | 2 | 4 | 3 | 1 | 3.5 | 3.5 | 1.5 | 1.5 |
| 6 | 3.5 | 3.5 | 2 | 1 | 3.5 | 3.5 | 2 | 1 |
| 7 | 4 | 1 | 3 | 2 | 4 | 2.5 | 1 | 2.5 |
| 8 | 4 | 3 | 1 | 2 | 2.5 | 2.5 | 2.5 | 2.5 |
